# Assessing Whether Measurement Invariance of the KIDSCREEN-27 across Child-Parent Dyad Depends on the Child Gender: A Multiple Group Confirmatory Factor Analysis

**DOI:** 10.5539/gjhs.v6n5p142

**Published:** 2014-05-19

**Authors:** Zahra Bagheri, Peyman Jafari, Elahe Tashakor, Amin Kouhpayeh, Homan Riazi

**Affiliations:** 1 Department of Biostatistics, Shiraz University of Medical Sciences, Shiraz, Iran; 2 Department of Pharmacology, Fasa University of Medical Sciences, Fasa, Iran; 3 Department of Vascular Surgery, Hannover Medical School, Hannover, Germany

**Keywords:** measurement invariance, children, parents, quality of life, KIDSCREEN-27

## Abstract

This study aims to assess the measurement invariance (MI) of the KIDSCREEN-27 questionnaire across girl-parent and boy-parent dyad to clarify how child gender affects the agreement between children’s and parents’ perception of the meaning of the items in the questionnaire. The child self-reports and parent proxy-reports of the KIDSCREEN-27 were completed by 1061 child-parent dyad. Multiple group categorical confirmatory factor analysis (MGCCFA) was applied to assess MI. The non-invariant items across girl-parent dyad were mostly detected in the psychological well-being and the social support and peers domains. Moreover, the boys and their parents differed mainly in the autonomy and parent relation domain. Detecting different non-invariant items across the girl-parent dyad compared to the boy-parent dyad underlines the importance of taking the child’s gender into account when assessing measurement invariance between children and their parents and consequently deciding about children’s physical, psychological or social well-being from the parents’ viewpoint.

## 1. Introduction

The importance of pediatric health-related quality of life (HRQOL) assessment to identify children being at risk of health problems has recently received much attention (Eiser & Morse, 2001a). Over the past two decades, an increasing number of well-known questionnaires have been developed to measure children’s HRQOL based on both children’s and parents’ point of views (Varni, Katz, Quiggins, & Friedman-Bender, 1998; Varni, Seid, & Kurtin, 2001; Reily et al., 2004; Ravens-Sieberer, 2001; Modi & Quittner, 2003; Parsons et al., 2005; le Coq, Boeke, Bezemer, Colland, & van Eijk, 2000). Nevertheless, agreement between self- and proxy- ratings continues to be a controversial issue in pediatric HRQOL research. It has been shown that child-parent agreement could be affected by child characteristics such as age and health status (Eiser & Morse, 2001b; Upton, Lawford, & Eiser, 2008; Buck, Clarke, Powell, Tiffin, & Drewett, 2012; Dey, Landolt, & Mohler-Kuo, 2013; White-Koning et al., 2007; Klassen, Miller, & Fine, 2006; Sattoe, Staa, & Moll, 2012).

However, the potential influence of child gender has been rarely assessed in the literature and no consistent results have been reported (Eiser & Morse, 2001b; Upton et al., 2008; Buck et al., 2012). For example, it has been shown that parents of boys with asthma and also atopic dermatitis had more optimistic perception of their child’s HRQOL compared to parents of girls (Chernyshov, 2012; Svavarsdottir, Burkhart, Rayens, Orlygsdottir, & Oakley, 2011). Moreover, a number of studies reported higher child-parent agreement among girls in all domains of the KIDSCREEN-52 (Robitail, Siméoni, Ravens-Sieberer, Bruil, & Auquier, 2007; Guedes, 2010). In contrast, other studies showed that only in some aspects of HRQOL, significant differences were observed between self- and proxy-reports among girls, while in other domains no gender effects were detected (Lundberg, Lindh, Eriksson, Petersen, & Eurenius, 2012; Theunissen et al., 1998).

However, in the literature cited above the agreement between child self-reports and parent proxy-reports was simply evaluated by comparing the mean of HRQOL scores. For a meaningful interpretation of mean differences, an essential assumption known as measurement invariance (MI) should be established (Meredith & Teresi, 2006; Teresi & Fleishman, 2007). MI shows that respondents from different groups interpret the concept of particular items similarly (Cheung & Rensvold, 2002; Vandenberg & Lance, 2000; Huang, Shenkman, Leite, Knapp, Thompson, & Revicki, 2009). If MI does not hold, it is not clear whether the observed disparity is a real difference in the underlying construct of interest or it is an artificial effect of different implicit or explicit interpretation of items by children and their parents (Cheung & Rensvold, 2002; Kim & Yoon, 2011). To the best of our knowledge, a limited number of studies have examined the MI of the pediatric questionnaires across child-parent dyad in different cultures (Huang et al., 2009; Lin, Luh, Cheng, Yang, Su, & Ma, 2013; Jafari, Bagheri, Hashemi, & Shalileh, 2013; Jafari, Sharafi, Bagheri, & Shalileh, 2013). However, one limitation of these studies is that assessing MI across children and their parents was conducted without taking the child’s gender into account. Hence, it has not yet become clear whether the agreement between children’s and parents’ perception of the items depends on the child’s gender. This issue is investigated in this study for the first time.

To explore MI, various statistical techniques are available, which have their own advantages and disadvantages (Kankaras, Vermunt, & Moors, 2011). Multiple group categorical confirmatory factor analysis (MGCCFA) is a method which can appropriately model the ordered-categorical responses, (Kim & Yoon, 2011), but rarely used in practice for pediatric HRQOL studies. For example, two of the mentioned studies (Huang et al., 2009; Lin et al., 2013) made use of the ordinary linear multiple group confirmatory factor analysis model (MGCFA) assuming that the response variable is continuous and normally distributed (Meredith, 1993).

The KIDSCREEN-27 is a well-known international generic HRQOL instrument with parallel child self-reports and parent proxy-reports. Although the MI of the KIDSCREEN have been established across different European countries (Revens-Sieberer et al., 2007; Robitail, Ravens-Sieberer, Simeoni, Rajmil, Bruil, & Power, 2007), this issue has not been evaluated across child-parent dyad yet. Therefore, this study aims to utilize the advantage of MGCCFA approach to examine the MI of the KIDSCREEN-27 across girl-parent and boy-parent dyad to clarify how child gender affects the agreement between children’s and parents’ perception of the meaning of the items in the questionnaire.

## 2. Method

### 2.1 Data and Measure

The target population was composed of 287551 school children aged 8-18 years and their parents in Shiraz, in the academic year 2011-2012. The participants were selected based on a two-stage cluster random sampling technique from the four educational districts of Shiraz, southern Iran. In the first stage, 6 middle and 7 high schools in each educational district were chosen randomly by cluster sampling. Within the selected 24 middle schools and 28 high schools, 40 and 50 classes were chosen respectively by stratified sampling. Finally, in each class a simple random sample of students was selected. Both child self-reports and parent proxy-reports of the Persian version of the KIDSCREEN-27 questionnaire and informed parent consent forms were distributed in each classroom by a trained researcher asking the students to take them home to their parents. About 75% of parents signed the informed consent and agreed to participate in the study with their children. Only one parent (mother or father) completed the proxy-reports, but there was no information on the gender of proxy rater in this study. The proxy-reports of the questionnaire were signed by parents to ensure that parents actually completed the questionnaire. All the children and their parents in our sampling population spoke Persian. They independently filled in the self- and proxy-reports at home. Finally 1061 completed child self-reports and parent proxy-reports of the questionnaire were returned to school by students. Of the 1061 children 593 (55.89%) were boys. The mean (± SD) age of boys was 13.65 ± 2.11 years and that of girls was 12.70 ± 2.65 years.

The KIDSCREEN is one of the international instruments for measuring HRQOL in children and adolescents. Both child self-reports and parent proxy-reports of this questionnaire are available in 3 versions including 52, 27 and 10 items (Erhart et al., 2009; Landgraf et al., 1998; Revens-Sieberer et al., 2007). Acceptable psychometric properties of the KIDSCREN-27 questionnaire have been confirmed in 13 European countries (Revens-Sieberer et al., 2007; Robitail et al., 2007). The KIDSCREEN-27 questionnaire was previously translated into Persian and its internal consistency and validity was assessed among Iranian school children (Jafari, Bagheri, & Safe, 2012). All domains in both child self-reports and parent proxy-reports had adequate internal consistency, i.e., Cronbach’s α coefficients were greater than 0.7. It ranged from α=0.73 (school environment) to α=0.85 (psychological well-being) and α=0.75 (school environment) to α=0.83(psychological well-being) in the self- and proxy-reports, respectively. In addition, the results of convergent and discriminant validity showed 100% scaling success rates in all domains. Confirmatory factor analysis also supported a five-factor model similar to its original version, and the results of Rasch analysis confirmed that all items belong to their own underlying construct (Jafari, Bagheri, & Safe, 2012).

This instrument encompasses five subscales of physical well-being (5 items), psychological well-being (7 items), autonomy and parent relation (7 items), social supports and peers (4 items), and school environment (4 items). All items were scored on a 5-point Likert scale from 1=never to 5=always or from 1=not at all to 5=extremely. For ease of interpretation, rating scale categories of negatively worded items were reversed so that higher scores showed better HRQOL.

### 2.2 Statistical Analysis

In this study, the MI of each domain of the KIDSCREEN-27 across girl-parent and boy-parent dyad was assessed by MGCCFA technique. An advantage of MGCCFA is that it can appropriately model the ordered-categorical responses by including the threshold structure (Kim & Yoon, 2011; Lubke & Muthen, 2004). Interested readers are encouraged to consult with Wirth and Edwards (2007) and Joreskog (2002) for exact mathematical description and technical information.

**Figure 1:**
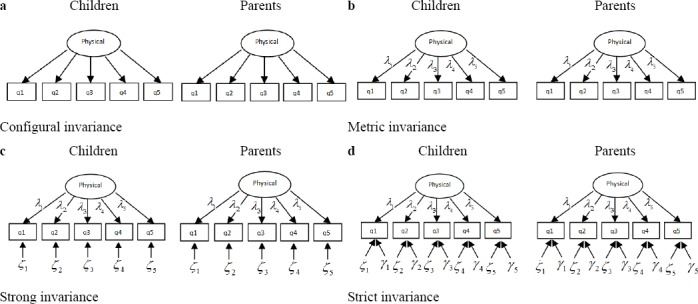
Four levels of measurement invariance for the physical well-being domain. (a) Configure invariance, (b) Metric invariance, (c) Scalar invariance, and (d) Strict invariance. Physical, factor score for the physical well-being domain; q, item score; *λ*, factor loading; *ζ*, intercept of item score; *γ*, residuals

In the present study, four levels of invariance including configural, metric, strong, and strict invariance were tested by setting four types of constraint on a set of parameters in an increasingly hierarchical order. Configural invariance investigates whether an underlying construct of interest is measured by the same set of items across two groups i.e., children and their parents use the same conceptual framework in their appraisal of underlying construct of interest (Meredith, 1993). In this situation, no equality constraint is imposed on the parameters across groups (Meredith, 1993). However, in both groups the same configuration of salient (non-zero) or nonsalient (zero) factor loadings are considered for the items (Ritchey, Frank, Hursti, & Tuorila, 2003). After establishing configural invariance, the next step is to assess metric invariance which is a prerequisite for testing higher levels of invariance (Cheung & Rensvold, 2002). Metric invariance is investigated by testing whether factor loadings of a construct are equal across groups implying that the strength of relations between a specific scale items and their corresponding underlying constructs are the same across the groups (Wu, Zhen, & Bruno, 2007). Strong invariance requires that besides factor loadings, the intercepts of like items are equal across subgroups. Equality of groups’ intercept shows that no systematic biases exist in the response of a group to the given scale items (Wu et al., 2007). Strict invariance is the most constrained model in which the variance of item residual should be equal across subgroups in addition to the intercepts and factor loadings. Strict invariance implies that scale items measure the latent construct with the same degree of measurement error in both groups (Wu et al., 2007). Figure 1 provides a visual aid to better understand four types of invariance explained here for one domain of the KIDSCREEN-27 instrument (physical well-being domain).

In this study the concept of partial measurement invariance is applied. When a certain level of invariance are not satisfied for all the items in a specific domain, the equality constraints on the parameters of the items demonstrating non-invariance between groups are removed one at a time and they will be allowed to be estimated freely (Byrne, Shavelson, & Muthen, 1989). Assessing partial invariance allows researchers to identify non-invariant or problematic items, which is the purpose of our study (Sass, 2011). We utilized the information from modification index to identify non-invariant items. Large magnitude of pertinent modification indices may be indicative of non-invariance. A modification index is the expected reduction in the value of the chi-square statistics when a fixed or constrained parameter is freely estimated (Diamantopoulos & Siguaw, 2000).

MI hypotheses are assessed by comparing two nested models. For instance, for testing strong invariance a model requiring equality constraints on factor loadings and intercepts is compared to the one just with equal factor loadings across groups. For assessing the fit of each model the chi-square (χ^2^) statistic, and for testing relative fit of two nested model, the change in it (∆χ^2^) can be used. However, this well-known statistics is not a practical test of model fit due to the detection of even trivial differences under large sample size (Cheung & Rensvold, 2002). Therefore, due to the relatively large sample size used in the study, although the value of χ^2^ (with df) and (∆χ^2^) were reported, other fit indices like comparative fit index (CFI), ΔCFI (change in CFI values of two nested model), and root mean square error of approximation (RMSEA) were considered for a final decision about accepting or rejecting the hypothesis of interest owing to their more reliable results. Because Cheung and Rensvold (2002) recommended these comparative fit indices are more robust to model complexity and variety of sample size. A nonsignificant ∆χ^2^, value of CFI≥0.95, ΔCFI≤0.01, and RMSEA≤0.06 can support acceptable model fit (Cheung & Rensvold, 2002).

In this study, LISREL 8.52 (Jorskog & Sorbom, 2003) was used to estimate MGCCFA model in a three-stage process. In step 1 using PRELIS, the thresholds of each latent response variable were estimated by pooling data from the two groups based on maximum likelihood method. PRELIS is a companion program that serves as a pre-processor for LISREL. It is used for calculating sample correlation and covariance matrices from raw data, and for estimating asymptotic covariances. In step 2, while holding the thresholds fixed at the previous step estimated values, the mean, asymptotic covariance matrices, and polychoric correlation were estimated for each group using conditional maximum likelihood. Finally, in step 3, the model parameters are estimated using generalized least square and MI hypotheses were examined (Muthen & Asparouhov, 2002; Jorskog, 2002).

## 3. Results

Table 1 shows the items identified with each type of measurement non-invariance across girl-parent as well as boy-parent dyad. No items were flagged as measurement non-invariant in the psychological well-being domain between boys and their parents and in the school environment domain in both boy-parent and girl-parent dyad. Tables 2 and 3 present the values of fit indices of MI for each domain of the KIDSCREEN-27 in girl-parent and boy-parent dyad, respectively.

**Table 1:** Measurement non-invariant items of the KIDSCREEN-27 across child-parents dyad

	Girls			Boys		

	Metric noninvariance	Strong noninvariance	Strict noninvariance	Metric noninvariance	Strong noninvariance	Strict noninvariance
**Physical well-being**						
q1. How would you say your health is					×	
q2. Felt fit and well			×		×	×
q3. Been physically active			×			×
q4. Been able to run well						
q5. Felt full of energy						
**Psychological well-being**						
q1. Your life been enjoyable		×				
q2. Been in a good mood	×					
q3. Had fun						
q4. Felt sad						
q5. Felt so bad that you didn’t want to do anything						
q6. Felt lonely						
q7. Been happy with the way you are	×					
**Autonomy and parent relation**						
q1. Had enough time for yourself					×	
q2. Been able to do thing					×	
q3. Your parent had enough time for you		×			×	
q4. Your parent treated you fairly		×			×	
q5. Been able to talk to your parent						
q6. Had enough money to do things as your friend					×	
q7. Had enough money for your expenses		×				
**Social support and peers**						
q1. Spent time with your friends		×	×			
q2. Had fun with your friends		×	×			
q3. You and your friends helped each other		×	×			
q4. Been able to rely on your friends		×	×			×
**School environment**						
q1. Been happy at school						
q2. Got on well at school						
q3. Been able to pay attention						
q4. Got along well with your teachers						

### 3.1 Configural Invariance

Configural invariance was supported by the values of CFIs≥0.95, RMSEAs≤0.06, and ∆CFIs≤0.01 for all domains across girl-parent and boy-parent dyad. This means that regardless of child gender, children and their parents used the same conceptual framework in their appraisal of all domains in the KIDSCREEN-27.

### 3.2 Metric Invariance

The fit indices values of metric invariance model were acceptable for all domains across boy-parent dyad. However, across girl-parent dyad, this hypothesis was supported for all domains except for the psychological well-being domain (RMSEA=0.063 and ∆CFI=0.011), in which the value of modification indices (not shown here) indicated that items 2 (Been in a good mood) and 7 (Been happy with the way you are) had different factor loadings among the groups. This result suggests that the associations of all items with their corresponding underlying constructs are equivalent across the groups except for the two cited items (items 2 and 7). After relaxing the equality constraints of the factor loadings of these two items across groups, partial metric invariance was accepted (RMSEA=0.054, CFI=0.968, and ∆CFI=0.001).

### 3.3 Strong Invariance

Moreover, the full strong invariance was held for the physical well-being and the school environment domains across girl-parent dyad (according to the acceptable values of CFA, ∆CFI, and RMSEA of Model 4 in Table 2) and for the psychological well-being, the school environment, and the social support and peers domains across boy-parent dyad (based on the satisfactory values of fit indices of Model 4 in Table 3). However, after removing the invariance constraints on the intercept of item 1 (Your life been enjoyable) in the psychological well-being domain and that of items 3 (Your parent had enough time for you), 4 (Your parent treated you fairly), and 7 (Had enough money for your expenses) in the autonomy and parent relations domain, the partial strong invariance was accepted across girl-parent dyad. Across boy-parent dyad, the modification indices suggested freely estimating the intercepts for items 1 (How would you say your health is) and 2 (Felt fit and well) in the physical well-being domain and for items 1 (Had enough time for yourself), 2 (Been able to do thing), 3 (Your parent had enough time for you), 4 (Your parent treated you fairly), and 6 (Had enough money to do things as your friend) in the autonomy and parent relations domain, resulting in well-fitting partial strong invariance. Non-invariant intercepts of the above items indicates that children and parents responded differently to these items.

**Table 2:** Assessment of measurement invariance for five domains of the KIDSCREEN-27 across girl-parent dyad

	Reference model	χ^2^ (d.f)	Δχ^2^	p-value	RMSEA	CFI	ΔCFI
**Physical well-being**							
M1: Configural invariance	-	16.18(6)	-	-	0.000	0.995	-
M2: Metric invariance	M1	32.67(9)	16.49	<0.001	0.028	0.989	0.006
M3: Partial metric invariance	-	-	-	-	-	-	-
M4: Strong invariance	M2	56.19(14)	23.52	<0.01	0.045	0.981	0.008
M5: Partial strong invariance	-	-	-	-	-	-	-
M6: Strict invariance	M4	132.20(19)	76.01	<0.001	0.072	0.934	0.047
M7: Partial strict invariance (q2, q3)	M4	83.78(17)	27.68	<0.001	0.050	0.971	0.010
**Psychological well-being**							
M1: Configural invariance	-	135.82(25)	-	-	0.055	0.970	-
M2: Metric invariance	M1	177.78(31)	41.96	<0.001	0.063	0.959	0.011
M3: Partial metric invariance (q2, q7)	M1	143.44(29)	7.62	0.106	0.054	0.968	0.001
M4: Strong invariance	M3	193.37(37)	49.93	<0.001	0.061	0.958	0.01
M5: Partial strong invariance (q1)	M3	160.69(35)	17.25	0.008	0.052	0.966	0.002
M6: Strict invariance	M5	215.37(42)	54.68	<0.001	0.048	0.955	0.011
M7: Partial strict invariance	-	-	-	-	-	-	-
**Autonomy and parent relation**							
M1: Configural invariance	-	89.55(25)	-	-	0.022	0.976	-
M2: Metric invariance	M1	140.11(31)	50.56	<0.001	0.041	0.966	0.010
M3: Partial metric invariance	-	-	-	-	-	-	-
M4: Strong invariance	M2	448.04(38)	307.93	<0.001	0.100	0.888	0.078
M5: Partial strong invariance (q3, q4, q7)	M2	193.10(35)	52.99	<0.001	0.054	0.950	0.016
M6: Strict invariance	M5	244.54(42)	51.44	<0.001	0.052	0.940	0.010
M7: Partial strict invariance	-	-	-	-	-	-	-
**Social support and peers**							
M1: Configural invariance	-	19.95(4)	-	-	0.051	0.987	-
M2: Metric invariance	M1	32.98(7)	13.01	0.004	0.059	0.977	0.01
M3: Partial metric invariance	-	-	-	-	-	-	-
M4: Strong invariance	M2	57.74(11)	24.67	<0.001	0.074	0.956	0.021
M5: Partial strong invariance	-	-	-	-	-	-	-
M6: Strict invariance	-	-	-	-	-	-	-
M7: Partial strict invariance	-	-	-	-	-	-	-
**School environment**							
M1: Configural invariance	-	9.78(5)	-	-	0.000	0.996	-
M2: Metric invariance	M1	18.49(8)	8.71	0.03	0.020	0.990	0.006
M3: Partial metric invariance	-	-	-	-	-	-	-
M4: Strong invariance	M2	21.65(12)	3.16	0.53	0.051	0.991	0.001
M5: Partial strong invariance	-	-	-	-	-	-	-
M6: Strict invariance	M4	49.36(16)	27.71	<0.001	0.037	0.966	0.025
M7: Partial strict invariance	-	-	-	-	-	-	-

**Table 3:** Assessment of measurement invariance for five domains of the KIDSCREEN-27 across boy-parent dyad

	Reference model	χ^2^ (d.f)	Δχ^2^	p-value	RMSEA	CFI	ΔCFI
**Physical well-being**							
M1: Configural invariance	-	88.91(9)	-	-	0.060	0.961	-
M2: Metric invariance	M1	90.79(13)	1.88	0.865	0.057	0.961	0.000
M3: Partial metric invariance	-	-	-	-	-	-	-
M4: Strong invariance	M2	144.99(18)	54.20	<0.001	0.072	0.945	0.015
M5: Partial strong invariance (q1, q2)	M2	94.88(16)	4.09	0.252	0.060	0.957	0.004
M6: Strict invariance	M5	190.61(21)	95.73	<0.001	0.071	0.960	0.003
M7: Partial strict invariance (q2, q3)	M5	121.13(19)	26.25	<0.001	0.056	0.949	0.008
							
**Psychological well-being**							
M1: Configural invariance	-	93.65(22)	-	-	0.043	0.977	-
M2: Metric invariance	M1	116.63(28)	22.98	0.008	0.047	0.973	0.004
M3: Partial metric invariance	-	-	-	-	-	-	-
M4: Strong invariance	M2	161.52(35)	44.89	<0.001	0.056	0.962	0.011
M5: Partial strong invariance	-	-	-	-	-	-	-
M6: Strict invariance	M4	201.40(42)	39.88	<0.001	0.050	0.955	0.007
M7: Partial strict invariance	-	-	-	-	-	-	-
							
**Autonomy and parent relation**							
M1: Configural invariance	-	185.11(27)	-	-	0.054	0.948	-
M2: Metric invariance	M1	228.59(33)	43.48	<0.001	0.059	0.938	0.010
M3: Partial metric invariance	-	-	-	-	-	-	-
M4: Strong invariance	M2	634.39(40)	405.80	<0.001	0.110	0.832	0.106
M5: Partial strong invariance (q1, q2, q3, q4, q6)	M2	636.75(35)	408.16	<0.001	0.060	0.935	0.003
M6: Strict invariance	M5	669.23(43)	32.48	<0.001	0.054	0.928	0.007
M7: Partial strict invariance	-	-	-	-	-	-	-
							
**Social support and peers**							
M1: Configural invariance	-	32.46(3)	-	-	0.034	1.000	-
M2: Metric invariance	M1	46.19(8)	13.73	0.018	0.046	0.990	0.010
M3: Partial metric invariance	-	-	-	-	-	-	-
M4: Strong invariance	M2	58.38(12)	12.19	0.016	0.060	0.980	0.010
M5: Partial strong invariance	-	-	-	-	-	-	-
M6: Strict invariance	M4	79.72(16)	21.34	<0.001	0.064	0.949	0.031
M7: Partial strict invariance (q4)	M4	63.77(15)	5.39	0.145	0.049	0.970	0.010
							
**School environment**							
M1: Configural invariance	-	36.65(5)	-	-	0.056	0.978	-
M2: Metric invariance	M1	47.63(8)	10.98	0.012	0.058	0.973	0.005
M3: Partial metric invariance	-	-	-	-	-	-	-
M4: Strong invariance	M2	58.38(12)	10.75	0.03	0.057	0.968	0.005
M5: Partial strong invariance	-	-	-	-	-	-	-
M6: Strict invariance	M4	79.27(16)	20.89	<0.001	0.052	0.956	0.012
M7: Partial strict invariance	-	-	-	-	-	-	-

### 3.4 Strict Invariance

The most constrained assumption, i.e., strict invariance was revealed according to the acceptable values of fit indices of Model 6 for the psychological well-being, the autonomy and parent relations, and the school environment domains across girl-parent dyad as well as boy-parent ones. In both girl-parent and boy-parent dyad, after relaxing the equality of the residuals variance of the items 2 (Felt fit and well) and 3 (Been physically active) in the physical well-being domain, partial strict invariance was verified (RMSEA<0.06, CFI≥0.95, and ∆CFI<0.01 for Model 7 in Tables 2 and 3). Partial strict invariance was held for the social support and peers domain across boy-parent dyad after freely estimating the residuals variance of item 4 (Been able to rely on your friends) across the groups. Moreover, across girl-parent dyad strict invariance of this domain was completely rejected. Therefore, there is adequate evidence that the above-mentioned items showing non-invariance residuals variance measure the latent construct with different degree of measurement error across children and their parents.

## 4. Discussion

This is the first study assessing MI of the KIDSCREEN-27 across girl-parent and boy-parent dyad separately. The findings revealed that the Persian version of the KIDSCREEN-27 is not an invariant measure neither across boy-parent nor girl-parent dyad.

Results showed that different non-invariant items were distinguished across girl-parent dyad as compared with boys-parent dyad. While the non-invariant items across girl-parent dyad were mostly detected in the psychological well-being, and the social support and peers domains, the boys and their parents differed mainly in the autonomy and parent relation domain. This discrepancy can be attributed to extensive hormonal fluctuation in teenage girls leading to more psychosomatic disorders and emotional disturbance as compared to boys (Viira & Koka, 2012). Moreover, girls and boys behave differently in their social relations due to higher and also more contradictory social expectations placed on girls than their male counterparts (Seiffge-Krenke, 1999), which may result in a higher level of disparity across girl-parent dyad in the social support and peers. On the other hand, boys have a higher tendency to be independent in their youth than girls leading to higher levels of disagreement among boy-parent dyad in the autonomy and parent relations domain (Bisegger, Cloetta, von Rueden, Abel, & Ravens-Sieberer, 2005).

However, it should be noted that these assertions cannot be made definitely without acknowledging parent’s gender. For instance, fathers encourage their sons to independence and autonomy more than their daughters (Seiffge-Krenke, 1999). Upton et al. (2008) asserted that sons and daughters have different relationships with each parent and also fathers and mothers have different perspective from their child HRQOL (Hill, Kondryn, Mackie, McNally, & Eden, 2003). Hence, further studies should consider the sex composition of the child-parent dyad on MI of HRQOL questionnaires across these two groups.

The previous studies evaluating MI of the other pediatric HRQOL instruments (PedsQL 4.0 and KINDL) across child-parents dyad did not consider child gender (Huang et al., 2009; Jafari, Bagheri et al., 2013; Jafari, Sharafi et al., 2013; Lin et al., 2013). However, they arrived at the same conclusion that children and their parents interpret some items differently. In this condition, the observed mean differences may reflect differences in the interpretation rather than true differences. Therefore, comparing between children’s and parents’ ratings of pediatric HRQOL is meaningless. Our study revealed that caution is warranted in comparing boys’ and parents’ ratings for all domains of the KIDSREEN-27, except for the psychological well-being and the school environment domains, which indicate full MI. Likewise, comparison between girls’ and parents’ ratings for all domains, except for the school environment domain, must be done with caution.

In this case, it is important to develop a new or revised version of the instrument with invariant items for meaningful cross-group comparisons. However, non-invariance of an item should not per se lead to its elimination, especially when the instrument has high convergent and discriminant validity (Sireci, 2011). Regarding the KIDSCREEN-27, our previous study showed that both child self-reports and parent proxy-reports of the questionnaire had high convergent and discriminant validity, and the results of Rasch analysis confirmed that all items belong to their own underlying construct (Jafari, Bagheri, & Safe, 2012). Similarly, in spite of detecting some non-invariant items in the Persian version of the PedsQL 4.0 across children and their parents (Jafari, Bagheri et al., 2013), the instrument has good psychometric properties including high convergent and discriminant validity in both child self-reports and parent proxy-reports versions (Jafari, Bagheri, Ayatollahi, & Soltani, 2012). Therefore, we believe that rewording or modification of the non-invariant items is a better choice than removing.

## 5. Limitations

Our study has some limitations that merit attention when interpreting our findings. First, the effect of clustered data (individual within district, school and class) has not been taken into account which may result in increased likelihood of finding measurement non-invariant items (French & Finch, 2010). Hence, we suggest applying hierarchical ordinal logistic regression model (French & Finch, 2010) or multilevel multiple-indicator multiple-cause (MIMIC) approach (Finch & French, 2010) in future studies to obtain more reliable and realistic results. Second, we assessed the MI across child-parent dyad classified only by the gender of the children and also our participants were apparently healthy school children. Hence, further in-depth studies are needed to evaluate the effects of additional factors such as children’s age or health status in addition to parents’ age, gender, mental, and physical health status on the MI of pediatric HRQOL questionnaires across these two groups. Finally, for comparability of our results with those of previous studies (Huang et al., 2009; Jafari, Bagheri et al., 2013; Jafari, Sharafi et al., 2013), we run the model separately for each domain. However, considering correlation between domains (Cheung, & Rensvold, 1999) could be changed our results principally.

## 6. Conclusion

In conclusion, detecting different non-invariant items across the girl-parent dyad compared to the boy-parent dyad underlines the importance of taking the child’s gender into account when assessing measurement invariance between children and their parents and consequently deciding about children’s physical, psychological or social well-being from the parents’ viewpoint. Therefore, future studies should replicate or extend our findings from the analysis of KIDSCREEN-27 to other pediatric HRQOL measures and also in other cultures to provide a questionnaire with measurement invariant items which is also stable regarding child gender.
